# Histopathological Spectrum of Non-Neoplastic and Neoplastic Lesions of Thyroid: A Descriptive Cross-sectional Study

**DOI:** 10.31729/jnma.5038

**Published:** 2020-11-30

**Authors:** Dilasma Ghartimagar, Arnab Ghosh, Manish Kiran Shrestha, Sushma Thapa, OP Talwar

**Affiliations:** 1 Department of Pathology, Manipal College of Medical Science, Pokhara, Nepal; 2 Department of Radiology, Charak Memorial Hospital, Pokhara, Nepal

**Keywords:** *anaplastic*, *follicular*, *goiter*, *papillary carcinoma*, *thyroiditis*

## Abstract

**Introduction:**

Thyroid gland lesions are the most common endocrine disorders encountered globally. Diseases of the thyroid gland present with either an alteration of hormone secretion or as an enlargement of the thyroid gland. The objective of the study is to find the frequency of different thyroid lesions.

**Methods:**

A descriptive cross-sectional study was conducted at Manipal Teaching Hospital, Pokhara from Jan 2005 to Jan 2020. Ethical approval was taken from the Institutional Review Committee (Ref: 330). Patients who had undergone thyroidectomy procedures for both non-neoplastic and neoplastic thyroid lesions were enrolled. Convenient sampling was done. IBM Statistical Package for Social Sciences version 21 and Microsoft Excel were used.

**Results:**

Out of 345 thyroidectomy specimens, 246 (71.3%) cases of non-neoplastic lesions, and 99 (28.69%) cases of neoplastic lesions were present. There were 54 males and 291 females with a male to female ratio of 1:5.4. The age ranged from 9 to 76 years with a mean age of 43.67 years. In non-neoplastic lesions, the predominant lesion was the colloid goiter with 205 (83.33%) cases followed by Grave's disease and lymphocytic thyroiditis with 14 (5.69%) cases each. In neoplastic lesions, papillary carcinoma was the commonest lesion with 56 (56.56%) cases followed by follicular carcinoma with 14 (14.14%) cases and follicular adenoma with 13 (13.13%) cases. There were also 9 (9.09%) cases of anaplastic carcinoma in neoplastic lesions.

**Conclusions:**

Colloid goiter and papillary carcinoma was the most commonly encountered non-neoplastic and neoplastic lesion with a female predominance. Rare tumors like anaplastic carcinoma, papillary carcinoma, and follicular carcinoma with anaplastic transformation were also encountered.

## INTRODUCTION

The thyroid gland is the largest of all endocrine organs which plays wide and vital physiological roles in the body and can be affected by a broad variety of diseases ranging from functional, immunologically mediated enlargements to neoplastic conditions.^1,2^ Thyroid gland lesions vary in incidence in relation to the geographical area, age, sex, dietary and environmental factors.^3^

Thyroid swellings are frequent and occur in 4% of the population aged between 30 and 60 years. Most of the thyroid swellings are benign while 10% to 20% of the thyroid swellings are malignant.^2^ Histological classification of thyroid lesions especially neoplastic conditions is essential for further therapy and prognosis.^4^

The objective of the study is to identify the histopathological spectrum of thyroid gland lesions and find out the frequency of non-neoplastic and neoplastic thyroid lesions with respect to variables like frequency, age, sex distribution, and various histopathological patterns.

## METHODS

A descriptive cross-sectional study was conducted in the Department of Pathology, Manipal Teaching Hospital, Pokhara from January 2005 to January 2020. Ethical approval was taken from the Institutional Ethical Review Board (Reference No: 330). The study included patients who had undergone thyroidectomy procedures for both non-neoplastic and neoplastic thyroid lesions. Convenient sampling was done. The sample size was calculated as,

$$\begin{array}{ll} \text{n} & \text{= }{\text{Z}}^{\text{2}}\times \text{p}\times \text{(1}-\text{p)}/{\text{e}}^{\text{2}}\\ & \text{= }{\left(1.96\right)}^{\text{2}}\times \{0.5\times (1-0.5)\}/{(\text{0.06)}}^{\text{2}}\\ & \text{= }267 \end{array}$$

where,

n = required sample size
p = prevalence taken, 50%
e = margin of error, 6%
Z = 1.96 at 95% Confidence Interval (CI)

Taking a 20% non-respondent rate, the final sample size was 320. However, a total of 345 cases were taken in the study. The study material consists of thyroidectomy specimens that encompass lobectomy, partial thyroidectomy, hemithyroidectomy, subtotal thyroidectomy, and total thyroidectomy specimens received from the departments of surgery and ear, nose, and throat (ENT). All the specimens were fixed in 10% buffered formalin and multiple sections from representative areas were taken. Detailed information regarding age, gender, duration of the lesion, side of the lesion along with relevant investigations like thyroid function test, and radiological reports if available were obtained. The slides were stained with routine hematoxylin and eosin (H&E) stain.

The data were analyzed using Microsoft Excel and Statistical Package for the Social Sciences 21.0 version.

## RESULTS

The study included 345 cases of thyroidectomy specimens collected over a period of 15 years. The study revealed 246 (71.3%) cases of non-neoplastic lesions and 99 (28.69%) cases of neoplastic lesions. Female predominance was recognized in both the non-neoplastic and neoplastic lesions of the thyroid. There were 54 males and 291 females with a male to female ratio of 1:5.4. The age ranged from 9 to 76 years with a mean age of 43.67 years.

Colloid goiter (CG) comprising of 205 (83.33%) cases was the dominant lesion among non-neoplastic lesions. Colloid goiter was affirmed in 181 cases of females and 24 cases of males with a male to female ratio of 1:7.5 (Table 1). Graves's disease and lymphocytic thyroiditis, accounted for 14 (5.69%) cases each, followed by Hashimoto's thyroiditis with 7 (2.84%) cases and female sex was predominant in all the lesions. No male involvement was noted in the case of Hashimoto's thyroiditis.

Tubercular involvement of the thyroid gland was reported in 4 cases with equal distribution in both the sexes (Table 1). All the cases showed well-formed epithelioid granulomas, multinucleated Langhan's giant cells and large areas of caseous necrosis. An abscess was observed in only one case of non-neoplastic lesion where the entire lobe of the thyroid showed neutrophilic collection with areas of fibrosis. A rare case of cysticercosis was divulged in a male patient who presented with a cystic swelling of the thyroid and microscopic examination revealed a fragment of cysticercus covered by corrugated chitinous layer with surrounding chronic inflammatory cells.

**Table 1:** Non-neoplastic lesions in relation to age and gender (n = 246).

Non-neoplastic lesion				Age-wise distribution							
	No of cases n (%)	Male	Female	0-11	11-20	21-30	31-40	41-50	51-60	61-70	>71
Colloid goiter	205 (83.33)	24 (9.74)	181 (73.57)	0	7 (2.84)	28 (11.38)	42 (17.07)	65 (26.42)	42 (17.07)	16 (6.50)	5 (2.03)
Grave's disease	14 (5.69)	4 (1.62)	10 (4.06)	0	2 (0.81)	2 (0.81)	5 (2.03)	4 (1.62)	0	1 (0.4)	0
Hashimoto's thyroiditis	7 (2.84)	0	7 (2.84)	0	1 (0.4)	1 (0.4)	3 (1.21)	1 (0.4)	1 (0.4)	0	0
Lymphocytic thyroiditis	14 (5.69)	1 (0.4)	13 (5.28)	0	0	3 (1.21)	6 (2.42)	2 (0.81)	2 (0.81)	1 (0.4)	0
Abscess	1 (0.4)	0	1 (0.4)	0	0	0	0	1 (0.4)	0	0	0
Tuberculosis	4 (1.62)	2 (0.81)	2 (0.81)	0	0	0	1 (0.4)	1 (0.4)	0	1 (0.4)	1 (0.4)
Cysticercosis	1 (0.4)	1 (0.4)	0	0	1(0.4)	0	0	0	0	0	0
Total	246 (100)	8 (3.25)	33 (13.41)	0	11 (4.47)	34 (13.82)	57 (23.17)	74 (30.08)	45 (18.29)	19 (7.72)	6 (2.43)

Colloid goiter was more prevalent on the right side than the left with secondary changes observed in 168 (82%) cases and a size ranging from 3 cm to 33 cm in maximum diameter. It was recognized predominantly in the 5th decade of life with 65 (31.7%) cases followed by the 4th and 6th decade of life, consisting of 42 (20.48%) cases each. Lymphocytic thyroiditis was witnessed in 11 (5.36%) cases of colloid goiter (Table 2).

**Table 2:** Colloid goiters in relation to age and gender (n = 205).

Age range	No of cases n (%)	Male	Female	Size range (cm)
0-10	0 (0)	0	0	0
11-20	7 (3.41)	1 (0.48)	6 (2.92)	3.5-18
21-30	28 (13.65)	2 (0.97)	26 (12.68)	4-15
31-40	42 (20.48)	6 (2.92)	36 (17.56)	6-26
41-50	65 (31.7)	6 (2.92)	59 (28.78)	3-25
51-60	42 (20.48)	5 (2.43)	37 (18.04)	6-26
61-70	16 (7.80)	3 (1.46)	13 (6.34)	5-24
>71	5 (2.43)	1 (0.48)	4 (1.95)	8-33
Total	205 (100)	24 (11.7)	181 (88.29)	

Follicular adenoma was the commonest benign neoplasm accounting for 13 (13.13%) cases. Female predominance was realized with 8 (61.53%) cases and age ranging from 16 years to 68 years with a mean age of 44.95 years. Hurthle cell adenoma was identified in 4 (4.04%) cases of thyroidectomy specimens with slight male preponderance and ages beyond 30 years of age. Papillary carcinoma (PC) thyroid was the prevailing malignant tumor with a total of 56 cases where the 49 (87.5%) female cases outnumbered the 7 (12.5%) males (Table 3). The age range for PC was 9 years to 74 years with a mean age of 42.06 years. Different subtypes of PC were witnessed where a follicular variant of PC was detected in 20 (35.71%) cases, a micropapillary variant of PC in two cases, and intracystic PC in one case. Lymphovascular emboli were determined in 22 (39.28%) cases of PC whereas capsular invasion and perineural invasion were noted in 9 (16.07%) and 3 (5.35%) cases respectively. Skeletal muscle invasion was discerned in 4 cases while skin infiltration was seen in 3 cases. Metastatic deposits in lymph nodes were ascertained in 5 cases. Bilobar involvement in 3 (5.35%) cases, PC in long-standing multinodular goiter 4 (7.14%) cases, and PC with associated Hashimoto's thyroiditis 3 (5.35%) cases were also established. There was only one case of anaplastic transformation in PC with lymphovascular embolization. Three cases of PC presented with skull metastasis and all were female patients above 50 years of age.

**Table 3:** Neoplastic lesions and its correlation with gender and age (n=99).

Types of tumors	No. of cases n (%)	Male	Female	Age-wise distribution							
				0-10	11-20	21-30	31-40	41-50	51-60	61-70	>71
Follicular adenoma	13 (13.13)	5 (5.05)	8 (8.08)	0	0	1 (1.01)	2 (2.02)	4 (4.04)	4 (4.04)	2 (2.02)	0
Hurthle cell adenoma	4 (4.04)	3 (3.03)	1 (1.01)	0	0	0	1 (1.01)	0	1 (1.01)	1 (1.01)	1 (1.01)
Papillary carcinoma	56 (56.56)	7 (7.07)	49 (49.49)	1 (1.01)	4 (4.04)	13 (13.13)	12 (12.12)	12 (12.12)	7 (7.07)	6 (6.06)	1 (1.01)
Follicular carcinoma	14 (14.14)	4 (4.04)	10 (10.1)	0	1 (1.01)	2 (2.02)	3 (3.03)	3 (12.12)	4 (4.04)	0	1 (1.01)
Medullary carcinoma	2 (2.02)	1 (1.01)	1 (1.01)	0	0	0	0	0	1 (1.01)	1 (1.01)	0
Anaplastic carcinoma	9 (9.09)	2 (2.02)	7 (7.07)	0	0	0	0	1 (1.01)	1 (1.01)	3 (12.12)	4 (4.04)
Lymphoma	1 (1.01)	0	1 (1.01)	0	0	0	0	1 (1.01)	0	0	0
Total	99 (100)	22 (22.22)	77 (77.77)	1 (1.01)	5 (5.05)	16 (16.16)	18 (18.18)	21 (21.21)	18 (18.18)	13 (13.13)	7 (7.07)

Follicular carcinoma (FC) was certified in 14 cases, age ranging from 23 years to 65 years with a mean age of 43.41 years and male to female ratio of 1:2.5 with 6th decade of life, in 4 (28.57%) cases, being frequent. The majority of the specimen had full capsular invasion excluding 3 (21.42%) cases which revealed minimal invasion of the capsule while 3 (21.42%) cases of vascular invasion and a case of lymph node metastasis were also observed. Extensive area of necrosis with anaplastic transformation was observed in one patient of FC. There was also a young patient of FC who presented with proximal arm swelling due to underlying bony metastasis and associated pathological fracture.

We observed only two cases of medullary carcinoma and the tumors were encapsulated in both the cases. Anaplastic carcinoma (AC) was detected in 9 (9.09%) cases involving 7 females and 2 males with a mean age of 65.33 years. Maximum numbers of AC were seen in the 8th decade of life with 4 cases followed by the 7th decade of life with 3 cases. The minimum age of the patient was 45 years while the maximum age was 76 years. All the cases presented with a neck mass with a mean duration of 5.6 months. The mean tumor size was 13.44 cm and the most common sub-type was the spindle cell type while two cases showed squamoid differentiation. All the cases of AC showed necrosis and mitosis >5/hpf. Muscular, neural and capsular invasions were seen in three, one and two cases respectively. Lymphovascular emboli and vascular malformation were noted in four cases each of AC while only one case had osseous metaplasia. B cell lymphoma was encountered in a 48 years old female with Hashimoto's thyroiditis. The tumor showed diffuse infiltration by neoplastic lymphoid cells along with few surviving thyroid follicles, focal areas of necrosis, and sclerosis. However, the 3 lymph nodes sent along showed reactive changes.

## DISCUSSION

Non-neoplastic and neoplastic thyroid lesions are common all over the world with varying prevalence and extent attributed to iodine deficiency and other environmental influences.^3^ In the present study, there was a significant number of non-neoplastic lesions as compared to neoplastic lesions constituting 246 (71.3%) and 99 (28.69%) cases respectively. Similar to our observation the incidence of non-neoplastic thyroid lesions as published in various studies has been reported to range from 62.5% to 84%.^1,3,4–7^

Colloid goitre is the prevailing lesions among the non neoplastic thyroid lesions with recorded incidence ranging from 56.93% to 76%.^1,3–5,7–9^ In the present study also, colloid goiter was the commonest non-neoplastic lesion in the 4th to 6th decade of life accounting for 205 (83.33%) cases. Singh SR and Iyengar S, Albasri A et al in their studies found the colloid goiter to be frequent in the 3^rd^ to 5^th^ decade of life.^6,7^ Some of the other non-neoplastic lesions in the present study include Grave's disease and lymphocytic thyroiditis with 14 (5.69%) cases each and Hashimoto's thyroiditis with 7 (2.84%) cases. The proportion of Grave's disease expressed in some of the research has been 0.3% and 2.5%.^5,6^ Lymphocytic thyroiditis was observed up to 1% and 15% in studies.^4,8^ While Hashimoto's thyroiditis was witnessed as high as 9.1% to 14%.^4,8,9^

Tuberculosis of the thyroid gland is an uncommon disease and more often seen in countries where tuberculosis is endemic.^10,11^ Kevin MC et al reported one case, Baidya A et al. from India reported two cases, and Majid U and Islam N from Pakistan reported 3 cases of tuberculosis of the thyroid gland.^10–12^ Similarly we stumbled upon 4 cases of thyroid tuberculosis in our survey. Thyroid abscess is a rare severe infectious condition of the thyroid gland with the staphylococci and streptococci species being the usual causative organisms responsible. We observed a case of thyroid abscess in a 45-year-old lady in the present research. Comparable to our findings Adeyemo A et al discovered a case of thyroid abscess in a 14-month-old child; however, Falhammar H et al identified 6 adults with thyroid abscess.^13,14^ Cysticercosis is a trivial tropical disease but its manifestation in the thyroid gland is scarce. Bothale KA et al. came across a case of cysticercosis in the thyroid gland while Bhalla A et al discovered a case of cysticercosis in 35 years old female who presented with generalized tonic-clonic seizures.^15,16^ We also report a case of cysticercosis in 19 years old male.

Among the benign neoplastic lesions, we recorded 13 (13.13%) cases of follicular adenoma with a maximum of 4 cases in the 5^th^ decade of life which was comparable to Fathima A et al. who disclosed 15 (12.5%) cases.^9^ The percentage of cases of follicular adenoma has been reported to be as low as 12.5% to as high as 56.67%.^4,7,8,17^ Present study revealed Hurthle cell adenoma in 4 individuals (4.04%). The reported percentage of Hurthle call adenoma in different publications is 2.46% (2 cases), 5.4% (6 cases), and 5.88% (12 cases).^6,8,17^

Papillary carcinoma was the most familiar neoplastic lesion in females of 3^rd^ to 5^th^ decades with 56 (56.56%) cases, which was analogous to that documented by Padmavathi M and Raj JA with 37 (58.73%) cases.^1^ The extent of PC spans from as low as 10% to as high as 72.97%.^6–9,17^ Our current analysis divulged distinct variants of PC which includes a follicular variant of PC, micropapillary variant of PC, intracystic PC with 20 (35.71%), 2 (3.57%), and 1 (1.78%) cases respectively. Urmiladevi P et al. in their review published 3 (15%) cases of follicular variant of PC and 2 (10%) cases of the micropapillary variant of PC.^4^ Padmavathi M and Raj JA in their analysis quoted 23 (62.1%) cases of follicular variant of PC and 2 (5.5%) cases of the micropapillary variant of PC.^1^ While Sreedevi AR and Sheela KM specified only about 7 (8.64%) cases of the micropapillary variant of PC.^8^ Baser B et al. has also specified about the PC arising within the cystic lesion in a 40 years old female where the mass gradually increases over a period of 6 months.^18^ In the present observation, PC was demonstrated in four cases of long-standing MNG. Urmiladevi P et al. and Memon W et al. have also come across PC in long-standing

MNG in one and 9 cases respectively.^4,19^ We also recognized a case of anaplastic transformation in PC in 74 years old female. Park JH et al. reported a case of anaplastic transformation of PC in 23 years old man while Abe T et al. recorded similar findings in a 61-year-old male.^20,21^ Thyroid carcinoma metastasis to the skull bone is rare and is found in only 2.5% to 5.8% cases of thyroid carcinoma. Li X et al in their study reported PC metastasis to the skull in 61 years old female.^22^ Tunio MA et al. also reported 3 cases of skull metastasis of PC in their study.^23^ We also came across 3 cases of PC metastasized to the skull bone.

We examined 14 (14.14%) cases of follicular carcinoma in our study with a peak incidence in the 4th to 6th decade of life. Padmavathi M and Raj JA, Sreedevi AR, and Sheela KM, Beigh A, et al, have disclosed lower percentages of FC in their reviews accounting for 2 (3.2%), 4 (3.60%), and 14 (6.86%), cases respectively.^1,8,17^ Anaplastic transformation of FC in metastatic sites was observed in 72 years old person by Lee W et al. while Nakayama R et al. also reported anaplastic transformation of FC in the skeletal metastatic site in 55 years old woman.^24,25^ We also uncovered a case of FC with anaplastic transformation in 45 years old female. Unlike the above-mentioned reports, we identified the anaplastic transformation in the primary site as opposed to the metastatic site. The frequency of metastasis in FC has been disclosed to be 6-20% with long-term survival rates ranging from 31% to 43%.^26^ Wu K et al. have disclosed 20 cases of follicular carcinoma with skeletal metastasis.^27^ Varadarajan VV et al have also described facial skeletal metastasis of FC.^28^ We also established a case of FC with bony metastasis in the left proximal arm in a 35 years old female. We had 2(2.02%) cases of medullary carcinoma thyroid in our current research which was similar to that presented by Singh SR & Iyengar S, Fatima A et al. and Beigh A et al. with 2 (2.5%), 8 (3.92%) and 1 (5%) cases respectively.^7,9,17^

Nine (9.09%) cases of anaplastic carcinoma were identified in 7 females and 2 male patients who were predominantly in the 5^th^ to 8^th^ decade of life in the present study. In comparison to other researches with 3.33%-6.25% of cases, we documented a higher percentage of cases, probably attributed to a longer duration of study of 15 years.^3,4,9^ Primary thyroid lymphoma accounts for 5% of all thyroid malignancies which originates from B cells and affects only the thyroid gland and regional lymph nodes. Diffuse B-cell lymphoma is considered the most common type accounting for 50-80% of all primary thyroid lymphomas. The incidence of thyroid lymphoma ranges from 1%-4.93% according to various studies.^29,30^ We came across only 1 (1.01%) case of lymphoma in 48 years old female patient with Hashimoto's thyroiditis.

The limitation of the study is that it was carried out in a single tertiary care hospital in the Western Region of Nepal; hence this may not represent the entire population of this province. Similar studies with a larger sample size should be conducted in multiple centers which would provide a clearer picture of the thyroid lesions in Nepal.

## CONCLUSIONS

The present study revealed a distinct female dominance in all thyroid lesions. Colloid goiter was the most prevalent non-neoplastic lesions with a peak incidence in 4^rd^ to 6^th^ decade of life, while papillary carcinoma thyroid was the most commonly encountered neoplastic lesion with a peak incidence in 3^rd^ to 5^th^ decade of life. Very uncommon lesions like anaplastic carcinoma, papillary and follicular carcinoma with anaplastic transformation, lymphoma were also encountered in the study.

## Conflict of Interest

**None.**
